# Magnitude and variability of structural brain abnormalities in neuropsychiatric disease: protocol for a network meta-analysis of MRI studies

**DOI:** 10.1136/ebmental-2020-300229

**Published:** 2021-04-13

**Authors:** Robert McCutcheon, Toby Pillinger, George Welby, Luke Vano, Connor Cummings, Xin Guo, Toni Ann Heron, Orestis Efthimiou, Andrea Cipriani, Oliver Howes

**Affiliations:** 1 Department of Psychosis Studies, Institute of Psychiatry Psychology and Neuroscience, London, UK; 2 Institute of Social and Preventive Medicine, University of Bern, Bern, Switzerland; 3 Department of Psychiatry, University of Oxford, Oxford, UK

**Keywords:** schizophrenia & psychotic disorders, adult psychiatry, depression & mood disorders, personality disorders, psychiatry

## Abstract

**Introduction:**

Structural MRI is the most frequently used method to investigate brain volume alterations in neuropsychiatric disease. Previous meta-analyses have typically focused on a single diagnosis, thereby precluding transdiagnostic comparisons.

**Methods and analysis:**

We will include all structural MRI studies of adults that report brain volumes for participants from at least two of the following diagnostic groups: healthy controls, schizophrenia, schizoaffective disorder, delusional disorder, psychotic depression, clinical high risk for psychosis, schizotypal personality disorder, psychosis unspecified, bipolar disorder, autism spectrum disorder, major depressive disorder, attention deficit hyperactivity disorder, obsessive compulsive disorder, post-traumatic stress disorder, emotionally unstable personality disorder, 22q11 deletion syndrome, generalised anxiety disorder, social anxiety disorder, panic disorder, mixed anxiety and depression. Network meta-analysis will be used to synthesise eligible studies. The primary analysis will examine standardised mean difference in average volume, a secondary analysis will examine differences in variability of volumes.

**Discussion:**

This network meta-analysis will provide a transdiagnostic integration of structural neuroimaging studies, providing researchers with a valuable summary of a large literature.

**PROSPERO registration number:**

CRD42020221143.

## Background

A wide range of neuropsychiatric disorders have been associated with alterations in regional brain volumes.^1–3^ Understanding whether regional patterns of structural abnormalities differ between disorders as opposed to representing a more general transdiagnostic disease process has major relevance for understanding the pathophysiology of neuropsychiatric disease.

In addition to studying differences in the mean size of regional brain volumes, recent studies have highlighted that the variability of regional volume size also differs between healthy controls and individuals with psychiatric disorders.^2 3^ In some disorders, relatively homogenous volumetric changes to specific brain regions are observed, whereas other regions display more heterogenous differences, suggesting that structural alterations may only be present within certain subgroups of the diagnostic category.

Meta-analysis has frequently been used in attempts to synthesise findings from the large number of studies of brain volumes. These analyses, however, typically only examine a single disorder.^2 3^ Network meta-analysis is an approach that is generally used for the comparison of efficacy across multiple health interventions, but can also be used to allow for the coherent synthesis of structural imaging studies across multiple disorders. Previous transdiagnostic meta-analyses have occasionally been reported. However, these meta-analyses either studied a restricted range of diagnoses^4^; or used an activation likelihood estimate approach, which does not allow for quantification of effect sizes and so preclude determination of whether one disorder displays a regional volumetric alteration greater in magnitude than another.^1^

In the current protocol, we describe a network meta-analysis of structural MRI studies across a wide range of neuropsychiatric disorders. The primary objective of the study is to quantify patterns of similarity and differences between disorders in terms of regional brain volumes. The secondary objective is to examine how patterns of variability of brain volumes differ across neuropsychiatric diagnoses.

## Methods and analysis

### Types of studies

All relevant published observational studies that use MRI to compare brain volumes in one neuropsychiatric disorder to another, or to controls will be identified by searching the relevant international scientific literature.

### Types of participants

The eligible population consists of individuals age 18 and over, of both sexes, with established diagnoses of any of the following disorders: schizophrenia, schizoaffective disorder, delusional disorder, psychotic depression, clinical high risk for psychosis, schizotypal personality disorder, psychosis unspecified, bipolar disorder, autism spectrum disorder, major depressive disorder, attention deficit hyperactivity disorder, obsessive compulsive disorder, post-traumatic stress disorder, emotionally unstable personality disorder, 22q11 deletion syndrome, generalised anxiety disorder, social anxiety disorder, panic disorder, mixed anxiety and depression. In addition, data from control groups will be extracted. These diagnoses encompass the vast majority of neuropsychiatric disorders in terms of lifetime prevalence, with the exception of substance use disorders.^5^ We have chosen not to include substance use disorders due to the difficulties in disambiguating the brain changes associated with the pathophysiology of addiction, and those that result from the direct effects of substance use.

Diagnoses should have been made using standardised diagnostic criteria such as the Research Diagnostic Criteria, Diagnostic and Statistical Manual of Mental Disorders, Third Edition (DSM-III), DSM-III-R, DSM-IV, DSM-5, International Classification of Disease, 10th Revision (ICD-10), ICD-11 or the comprehensive assessment of At-Risk Mental States.^6^ Study arms explicitly examining participants with comorbid psychiatric or physical health disorders will not be included. Uncertainty regarding study eligibility will be decided by discussion between authors.

### Outcome measures

For each study, we aim to collect the mean and SD of volumetric (in mm^3^ or cm^3^) or thickness (mm or cm) measurements for global and/or regional brain structures. Brain volumes examined will include: whole brain, whole brain white matter, whole brain grey matter, whole brain cerebrospinal fluid, amygdala, anterior cingulate cortex, accumbens, caudate, cerebellum, corpus callosum, frontal lobe, hippocampus, insula, lateral ventricle, pallidum, parahippocampal gyrus, parietal lobe, putamen, temporal lobe, thalamus and third ventricle. If reported separately, values will be extracted for both left and right hemispheres.

If only subregions of the above regions are reported (eg, frontal pole and medial frontal cortex are reported, but no overall value for frontal lobe is reported), then all subregions for the region in question will be combined. For volume measurements, the overall mean volume measure will be obtained by summing the subregion volumes, with SD being calculated according to standard propagation of uncertainty formula with the between region correlation assumed to be 0.7. For thickness measurements overall mean volume measure will be obtained by averaging the subregion thickness values, with SD being calculated according to standard propagation of uncertainty formula, with the between region correlation assumed to be 0.7, and subregions weighted according to their estimated volume as reported within the Desikan-Killany atlas.^7^

If both normalised and non-normalised volumes are reported, non-normalised volumes are preferred. If grey and white matter values are reported separately for a region, grey matter values are preferred. If both volume and thickness measurements (in mm or cm) are reported, volume measurements are preferred.

### Search strategy

The search strategy will include terms related to the study population, study type and main outcome. This search will extract studies from the following databases: Embase (Ovid interface), Medline (Ovid interface) and PsycINFO (Ovid interface). Hand-searching will also be performed to supplement electronic database searches; this will involve reviewing the reference lists of studies meeting our eligibility criteria.

#### Search term

((“magnetic resonance imaging” or MRI) and volume and (schizophren* or psychosis or schizoaffective or delusional or bipolar or depression or depressive or affective or autism or ASD or ADHD or “attention deficit” or anxiety or OCD or “obsessive compulsive” or PTSD or posttraumatic or 22q or velocardiofacial or “emotionally unstable” or “borderline personality”)).ab,kw,ti.

### Data extraction

Extracted information will be as follows: number of participants in each group, mean age, gender (% male), ethnicity (% black, white, other), psychiatric diagnosis including any comorbidities, age at illness onset, illness duration, psychotropic usage, method of measurement (volume vs thickness, automated vs manual), magnetic field strength, units of measurement, mean±SD of regions stated above.

Seven researchers will select the studies and extract the relevant information (XG, LV, TAH, CC, RM, GW) into a shared google sheet. If there is evidence of overlapping samples between studies, the study with the larger sample size will be used.

### Data synthesis

A qualitative synthesis of the collected data will also be presented. This will include summary tables showing the characteristics of the study population—demographics, diagnosis, age at illness onset, illness duration, medication use and duration of pharmacological treatment, and a Preferred Reporting Items for Systematic Reviews and Meta-Analyses flow chart.

### Pairwise meta-analyses

The principal summary measure will be the standardised mean difference (Hedges’ g) between diagnostic groups for the volumes of different brain regions.^8^

The secondary summary measure will be the coefficient of variation ratio. This is a measure of how variability differs between two groups while controlling for mean, and has been used in previous meta-analyses of brain structure to identify if there is evidence of subgroup phenomena within psychiatric disorders.^2 3 9^

We will perform direct meta-analyses for all pairs with ≥3 studies to obtain mean brain volume differences with their accompanying 95% CIs using a random effects model. Analyses will be carried out in the statistical programming language R (V.3.5.1) using ‘metafor’ (V.2.1–0).^10^ Visual inspection of the forest plots will be used to investigate the degree of statistical heterogeneity, alongside monitoring of τ (the estimated SD of random effects) and the I^2^ statistic. An I^2^ of less than 25% will be deemed to correspond to low heterogeneity, 25%–75% medium heterogeneity and greater than 75% high heterogeneity. To help visualise and assess the extent of heterogeneity, we will also include prediction intervals in all forest-plots.

Small study effects and publication bias will be assessed for each pairwise comparison by visual inspection of the contour-enhanced funnel plot and by performing Egger’s test of the intercept for meta-analyses comprising at least 10 studies.^11^

### Assessment of the transitivity assumption

In an attempt to ensure transitivity in the network, we will exclude studies examining paediatric patients, and exclude studies in which physical and psychiatric comorbidities are specifically studied.

Potential effect modifiers include age, gender and ethnicity. As such, we will examine if age, gender (% male) and ethnicity (% white) of participants are similarly distributed across the different diagnoses and health control populations.

### Network meta-analyses

If there is sufficient similarity between studies in terms of age, gender and ethnicity, we will conduct a random-effects network meta-analysis to synthesise our data. Network plots will be generated using the ‘netgraph’ function from the package ‘netmeta’,^12^ with each node representing a specific disorder, the size of the node being proportional to the number of studies used, and the thickness of the lines (edges) between nodes being proportional to the number of pairwise comparisons.

We will use a frequentist approach to network meta-analysis using netmeta in R (V.1.0–1). In order to allow for comparison across different scanners and measurement approaches, we will express volume differences between disorders as a standardised mean difference (Hedge’s g).

We will produce forest plots using ‘ggplot2’ (V.2.2.1), where the control group will be used as the reference. League tables will be created to display the relative degree of volume alteration for the various diagnostic groups using the ‘netleague’ function.

For each brain region, we will use the P-scores to rank diagnostic groups based on the corresponding degree of volume alteration. This will be done using the ‘netrank’ function. This method will allow us to rank the diagnostic groups on a continuous 0–1 scale for each outcome of interest: a higher P-score indicates greater degree of volume alteration. To summarise results across brain regions and disorders in a single diagram, we will produce a ‘Kilim plot’.^13^

### Assessments of heterogeneity and inconsistency

Heterogeneity of each network will be assessed by monitoring of τ and by plotting the prediction intervals for all comparisons versus placebo. Consistency of each network (ie, the agreement between direct and indirect evidence) will be evaluated using a global method (Q statistic) as well as a local method (back-calculation method using the ‘netsplit’ function).^14^

### Sensitivity analyses

The Enhancing Neuro Imaging Genetics through Meta-Analysis (ENIGMA) consortium has published several large scale syntheses of neuroimaging data.^15 16^ It is not straightforward to determine the overlap between these studies and previously published work, as a result a sensitivity analysis will be ran both including and excluding ENIGMA studies.

### Metaregression analyses

In addition to neuropsychiatric disorders, multiple other genetic and environmental factors also influence brain volumes. These include ageing and the use of psychotropic medications. We will therefore perform a meta-regression analysis to examine the relationship between study-level means of participant characteristics (gender, age, ethnicity (% white), illness duration, medication use) and differences in brain volumes, for each diagnosis versus a control population. Meta-regressions will be performed using the metafor function in R statistical software (V.3.5.3) and plots will be generated using ‘ggplot2’. We will only perform this analysis for diagnoses compared with controls in at least five studies.

### Risk of bias

Two independent reviewers will assess the quality of each study using a modified version of the Newcastle-Ottawa Scale for case control studies in which the exposure category is not considered due to its lack of relevance for imaging studies. This is the most appropriate scale given that observational studies are expected to predominate. Each study can receive a score from zero (low quality, high risk of bias) to six stars (high quality, low risk of bias). A threshold of ≥4 stars will be used to designate a high-quality study.

The ‘Confidence in Network Meta-Analysis’ (CINeMA) application will be employed to evaluate the credibility of findings from network meta-analysis.^14 17^ As part of the CINeMA evaluation process, a risk of bias assessment is required for each study with each study categorised as at low, unclear or high risk of bias, we will use the same threshold of ≥4 stars to classify studies as being at low or high risk of bias.

## Discussion

Structural brain abnormalities in neuropsychiatric disease have been studied in increasing depth over the past half century, with the number of studies increasing dramatically following the advent of MRI. While meta-analyses of individual disorders aid in the synthesis of this vast body of research, understanding how findings regarding one disorder relate to another remains a major challenge. In recent years studies have undertaken transdiagnostic attempts, but these involve smaller numbers of participants than a meta-analytic approach allows for or do not encompass as broad a range of disorders. This network meta-analysis provides a powerful approach to deriving a coherent understanding of brain abnormalities across neuropsychiatric disorders.

## Data Availability

All data relevant to the study are included in the article or uploaded as supplementary information. All data relevant are presented within the manuscript.
